# Loaded Questions: Internet Commenters’ Opinions on Physician-Patient Firearm Safety Conversations

**DOI:** 10.5811/westjem.2017.6.34849

**Published:** 2017-07-11

**Authors:** Christopher E. Knoepke, Amanda Allen, Megan L. Ranney, Garen J. Wintemute, Daniel D. Matlock, Marian E. Betz

**Affiliations:** * University of Colorado School of Medicine, Adult & Child Consortium for Outcomes Research & Delivery Science, Aurora, Colorado; † Chicago Medical School at Rosalind Franklin University of Medicine and Science, North Chicago, Illinois; ‡ Brown University, Alpert Medical School, Department of Emergency Medicine, Providence, Rhode Island; § Brown University, Emergency Digital Health Innovation Program, Providence, Rhode Island; ¶ Rhode Island Hospital, Injury Prevention Center, Providence, Rhode Island; || University of California, Davis, Department of Emergency Medicine, Davis, California; # University of Colorado School of Medicine, Department of Medicine, Aurora, Colorado; ** VA Eastern Colorado Geriatric Research Education and Clinical Center, Denver, Colorado; †† University of Colorado School of Medicine, Department of Emergency Medicine, Aurora, Colorado

## Abstract

**Introduction:**

Medical and public health societies advocate that healthcare providers (HCPs) counsel at-risk patients to reduce firearm injury risk. Anonymous online media comments often contain extreme viewpoints and may therefore help in understanding challenges of firearm safety counseling. To help inform injury prevention efforts, we sought to examine commenters’ stated opinions regarding firearm safety counseling HCPs.

**Methods:**

Qualitative descriptive analysis of online comments posted following news items (in May–June, 2016) about a peer-reviewed publication addressing when and how HCPs should counsel patients regarding firearms.

**Results:**

Among 871 comments posted by 522 individuals, most (57%) were generally negative toward firearm discussions, 17% were positive, and 26% were neutral/unclear. Two major categories and multiple themes emerged. “Areas of agreement” included that discussions may be valuable (1) when addressing risk of harm to self or others, (2) in pediatric injury prevention, and (3) as general safety education (without direct questioning), and that (4) HCPs lack gun safety and cultural knowledge. “Areas of tension” included whether (1) firearms are a public health issue, (2) counseling is effective prevention practice, (3) suicide could/should be prevented, and (4) firearm safety counseling is within HCPs’ purview.

**Conclusion:**

Among this set of commenters with likely extreme viewpoints, opinions were generally negative toward firearm safety conversations, but with some support in specific situations. Providing education, counseling, or materials without asking about firearm ownership was encouraged. Engaging firearm advocates when developing materials may enhance the acceptability of prevention activities.

## INTRODUCTION

More than 30,000 people die each year in the United States from firearm-related injuries,^1^ leading organizations to call for increased attention to firearm injuries as a preventable public health problem.^2^, ^3^ They recommend healthcare provider- (HCP) delivered discussions of firearm risks, based on evidence that such conversations may enhance home firearm safety behavior and reduce injuries.^4^, ^5^ In addition, in June 2017 the American Medical Association House of Delegates passed a resolution calling for collaboration with stakeholders to develop “state-specific guidance for physicians on how to counsel patients to reduce their risk for firearm-related accidental injury or death by suicide…”^6^ Several studies have demonstrated support for discussions about firearms in some circumstances, highlighting clinical situations in which firearm safety discussions may be effective prevention practice.^7^–^10^ Despite efforts occurring in some states to prohibit HCPs from discussing firearm risks with patients,^11^ recent court rulings have found such legislation an unconstitutional infringement on providers’ First Amendment right to freedom of speech.^12^

Nevertheless, firearm safety discussions are not widely or routinely integrated into healthcare encounters, and public opinion may vary about when and where such conversations are appropriate.^7^, ^13^, ^14^ Emergency department providers working with suicidal patients report discussing firearms and other lethal means in only a fraction of circumstances,^14^ partially due to fear of offending patients.^15^ “Cultural competence” of providers has been suggested as a means of increasing acceptability and implementation of firearm safety conversations.^16^ Still unclear are the full meaning of competence in this context and how best to increase competence among HCPs. Framing firearm discussions as “means safety” instead of “means restriction” could increase the acceptability and effectiveness of physician-patient discussions of suicide risk.^17^ Collaborations between firearm and public health groups also offer promise.^18^–^20^ Otherwise, many gaps remain in our understanding of how to make firearm safety conversations as effective and acceptable as possible.

Using online media, including social media or Internet “comment” sections, for qualitative research permits the inclusion of extreme perspectives that would be difficult to reach otherwise. The anonymity of online comments may enhance the comfort and frankness of users.^21^ Health communication specialists have used online media to examine attitudes about controversial medical topics.^22^–^25^ To our knowledge, no prior work has examined online commentary to better understand the debate over HCP counseling about firearm safety.

In this study, we therefore sought to examine the content of reader-submitted online comments about firearm safety conversations in healthcare practice. We recognized a priori that the individuals engaging in online debates are not representative of the larger population. However, understanding the beliefs of strongly opinionated subpopulations provides context critical to helping improve the acceptability and effectiveness of firearm safety discussions for use in the wider population.

Population Health Research Capsule

What do we already know about this issue?

Professional societies advocate that doctors should counsel patients about firearm safety, but social and political opinions on such conversations vary.

What was the research question?

To characterize the opinions of Internet commenters regarding doctor-patient firearm safety counselling.

What was the major finding of the study?

Most comments (57%) were against firearm safety counseling, but it was supported in specific clinical circumstances.

How does this improve population health?

Understanding the extreme viewpoints of a vocal minority can highlight challenges and opportunities to improve implementation of safety-oriented care.

## METHODS

### Study Design & Data Source

We used a qualitative descriptive study^26^ and followed recommended guidelines for reporting qualitative research.^27^ For our sample, we restricted our search to comments made about a single journal publication in an attempt to standardize the topic of online debate. “Yes, You Can: Physicians, Patients and Firearms”^11^ was a review publication by members of our team that described situations in which providers should consider asking and counseling patients about firearms. The article appeared online in Annals of Internal Medicine on May 17, 2016, with numerous online news reports following. We searched for eligible reports by reviewing both “news” and “blogs” results on the article’s Altmetric page, supplementing this with a Google search using relevant keyword combinations (e.g., “physician,” “firearm,” “gun,” “doctor”) and a 10-day range (May 15–25, 2016). We also searched major news sources and purposefully sought websites representing a variety of perceived political viewpoints, following findings suggesting that online commenters are more honest when they feel that they will be supported.^21^ To focus the content of the discussions to be analyzed, we excluded news items not directly reporting on the Annals article and those that did not allow for public comments (Figure). Comments were analyzed using Dedoose (v 7.1.3: SocioCultural Research Consultants, Los Angeles, CA). We repeated our search in February 2017 and did not find any additional articles or comments that fit our search criteria, as the news stories and the debate they generated appeared immediately following the original article’s publication.

The study team included diverse professional and research backgrounds and varying experiences related to firearms. These included prior firearm safety training, recreational target shooting and hunting, personal losses to suicide, and clinical care of patients at risk of suicide and/or with firearm injuries. The study team had no known prior relationships with any of the individuals whose comments were analyzed. All data came from publicly available sources, and no commenters were contacted. The project was deemed exempt by the Colorado Multiple Institutional Review Board.

### Analysis

We used a team-based analytic approach and established techniques.^28^–^33^ Analysis was completed in the fall of 2016. In the analysis of comments, each included independent coding by at least two team members of the team (A1, A2, A6). First, we categorized comments using a priori codes for apparent sentiments regarding doctor-patient firearm safety conversations (positive, negative, or neutral/unclear). Second, we used thematic analysis to describe codes emerging within and across categories. In both passes, team members maintained consistent contact, with regular meetings to adjudicate differences and review analytic memos. We synthesized the final codes into a core set of themes using our inductive and deductive toolkit^31^, ^32^ in consultation with all investigators.

## RESULTS

Our data included comments from items appearing on eight sites (*Bloomberg, Forbes, Fox News, Huffington Post, Medscape, MinnPost*, the *New York Times,* and the *Washington Post*; Figure) published May 16–19, 2016. There were 871 comments made by 522 unique user names/avatars; the number of commenters varied across sites (Figure). Most comments were posted close to the date of publication, with the latest posted on June 19, 2016. Among the user names, 242 (46%) were identifiable as male and 33 (6%) as female; 247 (47%) could not be classified. Most users (76%) posted one comment (range: 1–32; interquartile range: 1–1). Comments are quoted verbatim here, respecting the often informal or grammatically incorrect styles of writing used online.

### Themes

Most online comments appeared to view patient-physician discussions of firearms negatively (57%; vs. 17% positive and 26% neutral/unclear). Emergent categories were “Areas of agreement” and “Areas of tension,” with several themes identified within each.

### Areas of Agreement

Whether commenters were “for” or “against” HCP-delivered discussions of firearm safety, there were areas of general agreement and consensus among commenters (Table 1).

#### 1. Firearm safety conversations are appropriate when the patient presents risk of harm to self or others

This view was espoused even by commenters who otherwise opposed discussing firearms in clinical contexts. The pertinence of discussing firearms within the context of mental health problems was sometimes framed as an obvious, natural outgrowth of conversations related to depression, erratic behavior, or risk of committing violence against others.

“I feel they should only ask if they see signs of major aggression or depression in someone. They of course should look out for signs that there has been violence, or if they are signs of emotional or mental distress that may cause them to act out against theirselves or others.”

There was disagreement, at times with racist or other inflammatory language, about how to identify at-risk individuals.

“if these doctors are speaking with young black males they probably should mention gun violence. Otherwise discussing this topic would be a waste of time. The rest of America is capable of controlling themselves and we don’t typically act like animals.”

#### 2. Firearm safety conversations are acceptable as injury-prevention education for parents

Discussions surrounding secure storage of firearms in the home to prevent unintentional access and subsequent injuries to children were generally viewed as acceptable, particularly when framed in the context of other dangers.

“I routinely talk about safety with patients, not only gun storage, but also texting while driving and wearing helmets while skate boarding. Most patients appreciate the reminder.”

#### 3. Educate, don’t ask: Informational materials are acceptable

General educational materials and approaches, especially those endorsed by gun-use advocacy groups, were viewed favorably. Safety promotion conversations and materials were favored if they provided information about firearm risks alongside efforts to address other common dangers.

“Doctors need to have a gun safety pamphlet on the wall. Just like pool safety, bathroom safety, chemical safety, car safety, ticks and dogs. Subjects that they are not experts on but can impact the safety of a kid/family.”

These comments frequently noted that safety information could be universally provided to patients, irrespective of whether they own firearms. Some commenters expressed concern that entering information about firearm access into medical records could place patients at risk of privacy invasion.

“There’s absolutely nothing stopping your Dr. from handing you a brochure on Gun Safety...The PROBLEM is that Doctors are entering your answer about Gun Ownership into your (now computerized) Medical Records....and the Government wants access to those records.”

#### 4. Doctors are not knowledgeable about firearms or the culture of gun ownership

Many commenters cited their own extensive experience with firearms as evidence that HCPs would provide little value to them when discussing firearm access and use.

“I am a certified rifle and pistol instructor, a certified Range Safety Officer, and was also trained my dear old Uncle Sam while vacationing at beautiful Parris Island, SC., where I learned to shoot....bigger guns. My doctor is welcome to ask me anything he needs to learn”

Conversely, individual anecdotes about doctors who are firearm owners, active participants in associated communities, or otherwise knowledgeable about firearms suggested a relationship between a HCP’s perceived competence and a commenter’s willingness to discuss firearm safety. Yet a few commenters suggested that basic firearm safety counseling need not require significant knowledge or training on the part of providers.

“Lord woman, does a doctor really need to be a gun safety expert to tell you that you need to lock up your gun because your kid has suicidal ideations?”

### Areas of Tension

We identified four areas of conceptual disagreement between commenters with positive versus negative views of firearm safety conversations (Table 2).

#### 1. Are firearms a public health concern?

Individuals who believed that firearm injuries and violence are not within the purview of healthcare tended to argue that guns and health were distinct. Commenters endorsing the public health importance of firearms often compared them to other dangers that are addressed in clinical conversations, such as domestic violence and household hazards.

“If you own a gun, that’s fine, be an adult about it and recognize that it *is* a health risk. I’m glad doctors are being persistent about this.”

#### 2. Is physician counseling effective in preventing injuries/deaths?

A positivistic view of research^34^ surrounding prevention methods, in which a lack of evidence of efficacy is viewed as evidence of inefficacy, was common.

“FACT: Any doctor that ask their patients about guns are quacks.”

Some commenters emphasized their own skepticism about approaches to firearm safety discussions without acknowledging existing studies supporting the effectiveness of firearm safety counseling.^4^, ^5^ Others offered hypothetical situations in which such conversations might be a low-risk, but potentially high-reward, injury/violence prevention strategy.

#### 3. Is suicide preventable? Should suicide be prevented?

Many commenters said that suicide was not preventable and that suicidal individuals without firearm access would substitute a different fatal method. Some specified that suicide is a reasoned action, and that reducing firearm access therefore infringed on individuals’ liberty to end their own lives.

“Why do the meddling do-gooders want to prevent suicides, if a person wants to end his life? The reason why older White males have a high suicide rate is because they are determined and decisive. (This is also the reason why most good executives are White males - they make decisions and get things done).”

Most arguments supporting suicide’s preventability were made by commenters who endorsed a positive view of firearm safety discussions. Many of these comments were written by individuals self-identifying as HCPs.

#### 4. Is firearm safety within HCP’s professional role?

Some commenters indicated that conversations with providers who lack requisite knowledge and cultural competence would be unhelpful and unnecessarily contentious. Others highlighted the intimate nature of other clinical conversations, situating firearms among myriad sensitive topics discussed within healthcare encounters.

“Doctors routinely advise patients not to drive because their age, vision, neurological problems, etc. make it a risk to themselves and others. They advise them not to work with certain kinds of machinery for similar reasons. Why shouldn’t docs advise patients on guns?”

### Other Themes

Additional notable themes were identified among negative comments.

#### 1. Firearm discussions are part of a hidden agenda for gun control

Distrust of medical and public health professionals was frequently coupled with commenters’ belief that discussions regarding firearm access were gathering data to support gun control efforts.

“Doctors are likely starting to be mandated to ask these questions and mine data for big brother.”

#### 2. Comparison to other health hazards

Risk attributable to firearms as compared with other health concerns was mentioned frequently.

“Sure, but odd you never hear about Doctors wanting to talk safety about the 28,000 chain saw injuries per year, those killed by open dishwashers, or any of the myriad other safety issues, but guns by golly, that’s the one they need to give you a colonoscopy on.”

Many commenters noted dangers and deaths associated with medical errors and prescription medication misuse (with varying statistics quoted), supporting the notion that HCPs are not competent to counsel patients about safety in any context.

“98,000 people die from doctor mistakes per year. And gunshot wounds a little more than 30,000 per year. Think about that.”

“The medical profession is responsible for 600,000+ unnecessary death each year, must we have a conversation with the NRA about seeking medical attention?”

## DISCUSSION

While the opinions of Internet commenters are certainly not representative of the general population’s opinions regarding firearm safety conversations, the extreme views expressed by this vocal minority offer unique insight into the perspectives of some who most vigorously oppose firearm safety discussions in a clinical context. A better understanding of these strongly held opinions could inform the strategies public health professionals use to implement prevention programming and providers’ decisions about how to frame firearm safety conversations. It also supports future hypothesis-guided research on best practices for such conversations.

The majority of commenters agreed on the appropriateness of three aspects of patient-provider firearm safety conversations: (1) counseling and intervention with individuals posing risk to themselves or others; (2) counseling parents; and (3) including educational materials in these discussions, especially materials created in collaboration with firearm advocacy organizations. These areas of agreement highlight possibilities for collaborations among public health professionals, HCPs, firearm organizations, parenting groups, violence prevention advocates, civil society advocates, and other stakeholders.^16^–^19^ Such collaborations could improve the quality and effectiveness of firearm safety discussions.

A key finding was that commenters viewed asking about gun ownership as different from educating about gun safety. This finding provides context to a recent survey of parents, in which slightly more supported counseling about safe firearm storage than asking about access (75% versus 66%).^9^ Providing information about firearm safety without inquiring about access and without singling out firearms as source of high risk was viewed favorably by online commenters, irrespective of the clinical context. Conversations about firearms could be added to those covering household hazards and prescription medications.

Many comments revealed misinformation or stigma about the preventability of suicide, highlighting the importance of efforts to educate providers and the public about the preventability of suicide. In circumstances where the patient poses an immediate risk to self or others, asking directly about firearm access is an evidence-based component of a physician’s risk assessment and determination of care. Yet, in a national survey 74% of respondents thought most or all suicide decedents would have found another way to die, had their chosen method been blocked; and HCPs still report skepticism about the preventability of suicide. Our findings similarly reflect lack of familiarity with the large body of evidence on the effectiveness of lethal means restriction (temporary reduction in access to highly lethal methods of suicide) as a suicide prevention approach.^35^, ^36^

Others’ work shows that high proportions of physicians believe they have a right and responsibility to talk to patients about firearm safety. Physician counseling about firearm safety is effective in changing home storage behavior in many circumstances,^5^, ^35^, ^37^ and a recent survey of firearm-owning parents found that 14% of parents would follow, and 49% would consider, a pediatrician’s advice to not have firearms in the home.^9^ Yet physicians are reticent to initiate these conversations in practice. Perceived barriers include low perceived efficacy, lack of confidence in their own credibility and purview, and concern that such conversations will alienate patients.^10^ Culturally competent materials on firearm safety discussions may help to overcome concerns about physician knowledge or trustworthiness. Educational materials created in collaboration with firearms groups may have greater credibility and acceptability to patients who own firearms. Indeed, some commenters in our data (who self-identified as HCPs) reported that they use materials from the National Rifle Association or National Shooting Sports Foundation, and materials are also available from other organizations.^38^, ^39^ Other ways to increase physician competence may include training, collaboration with firearm organizations, and improved awareness of state and local laws.

## LIMITATIONS

Limitations of this project include that the results are derived from discussions occurring between small sectors of generally anonymous online commenters, who are different than the general public in a variety of ways. People who comment online are more male and have lower educational attainment than people who read comments, but do not participate in discussions.^40^ Internet comments sections are likely to represent more extreme or hyperbolic views than those appearing in other public discourse.^41^ Online dialogue can be highly contentious, with many commenters defending positions they believed concordant with their global views on gun rights or control.

Unfortunately, we were not able to examine comments according to demographic characteristics, as gender was often unclear and other relevant factors (including age, race, and geographic area) were not available. Tension during the study period (May–June 2016) may have been heightened by partisan conversations following major news events and the U.S. presidential election. Indeed, many of the more extreme views arose in comments about gun control in general, rather than how to address firearm safety within healthcare. Prior studies suggest that many firearm owners have more positive views towards physician engagement in firearm safety than was illustrated in this analysis.^7^, ^9^ On the other hand, while our results likely describe a subpopulation with strong, extreme opinions, the online forum does have the advantage of anonymity and thus may be effectively bypassing social acceptability bias and uncovering more honest opinions. In addition, our focus on news stories and comments about a single published article allows us to control for some variability in the subject matter.

## CONCLUSION

This qualitative analysis of online comments about an article on HCP firearm safety discussions likely represents extreme views due to anonymity and the requisite motivation to engage in highly politicized conversation. Despite this, even some commenters with reservations about such discussions appeared to support them in particular circumstances. These circumstances, including counseling for those at risk to self or others, for parents, and with educational materials, echoed many of the primary points of the referenced article.^11^ The other area of agreement – tthat HCPs generally lack knowledge and cultural competency regarding firearm ownership – is one of the barriers reported by physicians themselves to expanding firearm safety counseling. Future research to better understand the most effective messages and methods for discussions about firearm safety is critical. In the meantime, providers can use culturally competent approaches and existing guidelines and recommendations – enhanced by a growing understanding of the views of those skeptical or opposed – to help prevent firearm injuries and deaths.

## Figures and Tables

**Figure:**
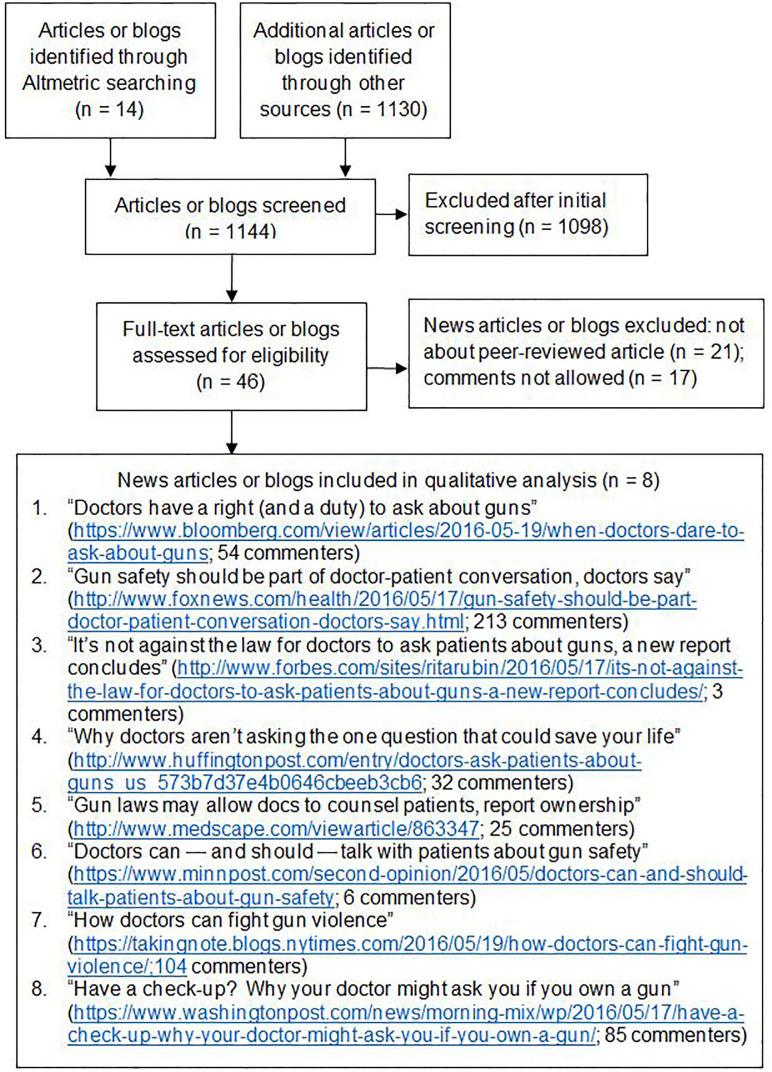
Flowchart of search strategy.

**Table 1:** Areas of agreement, with representative quotes.

Area	Quote
1. Firearm safety conversations are appropriate when the patient presents risk of harm to self or others	“Crisis/emergency intervention? Absolutely, yes. Ask and intervene. And don’t limit it to just guns, but other aspects where the patient presents a frank danger to themselves and others.” “If someone appears distraught or seems to be a threat to themselves, or someone else, then it makes sense to ask.”
2. Firearm safety conversations are acceptable as injury prevention education for parents	“The American Academy of Pediatrics isn’t recommending anything that the NRA isn’t already recommending. . . . We actually don’t care if you own a gun and do not share that information. We are only concerned if there are others in the house that could harm themselves or others if they have access to the weapon. . . . .” “It should go something like this…’Remember children are very inquisitive....store harmful liquids high and in a lockable cabinet, keep sharp objects, or hot pots and pans out of their reach, store firearms without ammunition and try to use a trigger lock or a safe while storing firearms. And remember to secure your child in a car safety seat while driving, use an approved seat and have it installed properly. Of you have a pool ensure that the pool is not accusable to your child unless you are present’.”
3. Educate, don’t ask: Informational materials are acceptable	“Okay, talk about them all you want. Discuss storage methods, explain how to keep them as safe as possible. Just don’t ask if the patient owns any and we’ll never have a problem.” “Confidential questions about gun habits, like questions about driving, smoking and drinking, are legitimate medical inquiries.” ASKING is not the problem, it is the SHARING with the Government/ THIRD PARTIES that becomes the issue!”
4. HCPs are not knowledgeable about firearms or the culture of gun ownership	“I have ONE doctor that I discuss firearms with and that is only because he is actually carrying during my visit. I hate to admit it, but he actually shoots better than I do too! The rest of my doctors have no business asking about my means of self defense or how many firearms I own. “

**Table 2:** Areas of tension, with representative quotes.

Question	No	Yes
1. Are firearms a medical/public health issue?	“It’s none of your business. Simply put. It’s not medicine, no matter how you try to stretch it.” “What EXACTLY, has been the contribution to “patient health” from physicians learning about the ownership of firearms?”	“If doctors can tackle domestic violence, why not gun violence? Both have been public health emergency conditions for years. I am not anti-gun. My family are all gun-owners, we all learn to shoot safely and get our own .22 when we turn 12.” “As the third-leading COD (second-leading in 2014), it is absolutely a medical issue, as are Cancers (first) and MVA’s (second).”
2. Is counselling effective in preventing injuries/deaths?	“I am a physician: I am trained to practice “evidence based medicine”. There is no evidence that this policy would help, and it cannot be seen as the practice of medicine” “Did Goebbels spring from his grave and pen this? What, EXACTLY, are physicians to do if the patient says, ‘Yes, I own a firearm.’ What EXACTLY, has been the contribution to “patient health” from physicians learning about the ownership of firearms?” “I’m all for doctors asking whatever questions they deem fit, but realistically how many lives are going to be saved? My guess is ‘very few’ to ‘none’. How many irresponsible gun owners are regularly visiting the doctor? How many of them would actually take on gun advice from said doctor? How many of those few who actually took the advice would then go home and follow through? If you want to curtail gun violence you only have one sure-fire method for doing so: start banning guns.”	“If Mrs. Lanza’s [mother of Adam Lanza the Newtown killer] physician had inquired about guns and said given your son’s mental condition it would be wise not to have guns in your home perhaps the Newtown massacre would have been prevented” “It’s not the gun safety issue, it’s the mental state of the owners issue the doctor should be watching for. My dad had a stroke and the doctor revoked his drivers license until he was well enough again and got it reinstated. Same if a doctor sees a patient is getting into a depressive state from a divorce or job loss etc, revoke the firearms license until they are better.”
3. Is suicide preventable? Should It be prevented?	“If someone is going to take their life then they will do it by any means necessary. They do not need a gun.” “The great majority of annual gun deaths are suicides in the middle-aged to elderly. Why do we think we need to prevent this? Who can say that this is not a rational decision for many of these people? Often it is a blessing for their families that they no longer have to deal with the intractable problems associated with living with or around these broken people.”	“if you were depressed and killed yourself with a gun and your family came after me to sue, I’m sure my malpractice insurer would be quite interested in whether or not I’d asked about guns” “What doctor doesn’t talk to suicidal patients about removing firearms from the home? I’ve done it countless times.”
4. Is firearm safety within HCPs’ professional role?	“Please. Like some ‘doctor’ knows what is and isn’t good for me.” “Safety is a lie told to stupid people to keep them in line...Doctors are not working for our benefit.” “If I want to see a doctor, it’s because I need medical attention, not because I need to get into a debate with a gun control advocate.”	“A lot of people who take gun freedom to gun nuttery seem to think that they need to get the government to pass a nanny-state law telling your doctor how to treat you because they don’t want to face an uncomfortable question.” “The point being is that doctors should be able to ask questions whenever they feel the need, and honestly? They shouldn’t have to spend most of the visit justifying the questions.”

**Table 3:** Other themes, with representative quotes

Theme	Quote
1. Belief that firearm discussions are part of a hidden agenda for gun control	“Doctors asking patients about owning guns during a simple check up is nothing but a new tactic by a segment of the gun-control crowd (anti-gun doctors) used in an attempt to stigmatize guns. Period” “Doctors asking about guns in the home seemed to become a phenomenon that started happening once the government took over the healthcare system. Doctors are likely starting to be mandated to ask these questions and mine data for big brother.”
2. Comparison to other hazards, often with inaccurate quotation of statistics to reinforce points of view	“It would be more appropriate for doctors to ask parents of small children it they have stairs in their house. Much higher injury, and fatality, rate from stairs. But stairs aren’t a political issue” “Right so should doctors routinely inquire about whether their patients own a motorcycle? How about extreme sports; do you rock climb? If so are we relying on doctors to provide instructions on safe riding practices or how to properly tie off, on a cliff?”
